# Follow-Up of PRRSv-Vaccinated Piglets Born from PRRSv-Vaccinated, ELISA-Seropositive and ELISA-Seronegative Sows

**DOI:** 10.3390/v15020479

**Published:** 2023-02-09

**Authors:** Jorian Fiers, Marylène Tignon, Dominiek Maes, Ann-Brigitte Cay

**Affiliations:** 1 Unit Viral Re-Emerging, Enzootic and Bee Diseases, Department Infectious Diseases in Animals, Sciensano, Groeselenbergstraat 99, 1180 Ukkel, Belgium; 2 Unit of Porcine Health Management, Department of Internal Medicine, Reproduction and Population Medicine, Faculty of Veterinary Medicine, Ghent University, Salisburylaan 133, 9820 Merelbeke, Belgium

**Keywords:** PRRSv, vaccination, immunology, maternally-derived antibodies, seroconversion

## Abstract

Vaccination against the porcine reproductive and respiratory syndrome virus (PRRSv) is widely used to prevent production losses in the swine industry. In this study, piglets born from both PRRSv-vaccinated ELISA-seropositive sows (E+ piglets) and PRRSv-vaccinated ELISA-seronegative sows (E− piglets) were followed-up pre-vaccination, 3 weeks post-vaccination (wpv) and 8 wpv in two Belgian farrow-to-finish herds. The aim of the study was to analyze the presence of PRRSv-specific maternally-derived antibodies (MDAs) and the PRRSv vaccine response in both groups of piglets. The E− piglets lacked the presence of PRRSv-specific MDAs (0% seropositive), while these were present in the E+ piglets (97% seropositive). Due to this, the E− piglets showed a strong initial vaccine response (72–80% seroconversion) and vaccine viremia (65–75% PCR positive) at 3 wpv. In contrast, the E+ piglets showed only limited initial vaccine responses (25–61% with increased ELISA values) and vaccine viremia (30–31% PCR positive) at 3 wpv. By 8 wpv, the proportion of seropositive E− piglets (78–100%) and seropositive E+ piglets (55–90%) increased in both herds. However, a difference in vaccine viremia duration was observed between both herds at 8 wpv, with a decrease in the proportion of PCR positive piglets in herd 1 (E−: 47%; E+: 25%) and an increase in the proportion of PCR positive piglets in herd 2 (E−: 85%; E+: 92%). This study identified clear differences in the presence of PRRSv-specific maternally-derived antibodies and PRRSv vaccine responses between E− and E+ piglets. Further research is warranted to elicit the biological relevance of these observed differences.

## 1. Introduction

The porcine reproductive and respiratory syndrome virus (PRRSv) is one of the most important pathogens in the swine industry, causing enormous production and economic losses [1,2]. This small, enveloped RNA virus belongs to the order *Nidovirales*, family *Arteriviridae* and is classified into two distinct species: *Betaarterivirus suid 1* (PRRSv-1) and *Betaarterivirus suid 2* (PRRSv-2) [3]. Clinical signs of PRRSv infection vary from reproductive failure in gilts and sows to respiratory distress and consequently growth retardation in growing pigs [4]. PRRSv vaccination of the sow and/or piglet population is widely practiced to control and prevent production losses caused by the virus. Two classes of PRRSv vaccines are commercially available: modified live vaccines (MLVs) and the inactivated/killed vaccines (KVs). Unfortunately, the effectiveness of PRRSv vaccination is rather limited, with PRRS outbreaks occurring in herds that practice routine vaccination [5,6,7,8]. Additionally, the use of PRRSv MLVs has some safety concerns, including the possible reversion to virulence and the emergence of recombinant PRRSv strains [9,10,11,12,13,14].

In a previous cross-sectional field study, our research group assessed the prevalence of multiple PRRSv-vaccinated, but ELISA-seronegative, sows in Belgium [15]. In that study, the presence of PRRSv-specific ELISA antibodies (Abs) was analyzed in 1400 PRRSv-vaccinated sows originating from 70 Belgian sow herds. Overall, 3.5% to 4.1% (depending on the ELISA kit used) of the sampled sows tested seronegative in ELISAs. Additionally, 40% of the sampled herds had at least one ELISA-seronegative sow (of 20 sows sampled). Based on this study, a follow-up study was designed to analyze the possible consequences of ELISA-seronegative sow status for its progeny. The first aim of this follow-up study was to investigate the presence of PRRSv-specific maternally-derived antibodies (MDAs) in piglets born from both PRRSv-vaccinated ELISA-seropositive and PRRSv-vaccinated ELISA-seronegative sows. It can be hypothesized that piglets born to the ELISA-seronegative sows receive no PRRSv-specific MDAs compared to piglets born to the ELISA-seropositive sows, who do receive PRRSv-specific MDAs. A second aim of this follow-up study was to analyze the PRRSv vaccine responses in both groups of piglets. If the former hypothesis is true, then it can be hypothesized that a different PRRSv vaccine response would be seen in both groups of piglets. Previous studies have already shown the interference of maternally-derived neutralizing antibodies (MDNAs) on PRRSv vaccine responses in vaccinated piglets [16,17]. If piglets born from the ELISA-seronegative sows receive no MDAs, then it could be hypothesized that these piglets would have a stronger vaccine response compared to the piglets born from ELISA-seropositive sows. Alternatively, if the observed ELISA non-responsiveness is a genetic characteristic, then it might be possible for the piglets born from the ELISA-seronegative sows to react less to the PRRSv-vaccination as well. To our knowledge, this was the first study in which PRRSv vaccine responses were compared between piglets born from PRRSv-vaccinated ELISA-seropositive and PRRSv-vaccinated ELISA-seronegative sows. Previous studies have focused on the comparison of PRRSv vaccine responses in piglets with high or low amounts of MDNAs [16,17]. 

## 2. Materials and Methods

### 2.1. Study Design

The study design was approved by the Ethical Committee of the Faculty of Veterinary Medicine and the Faculty of Bioscience Engineering of Ghent University under approval number 2021-037. 

**Figure 1:**
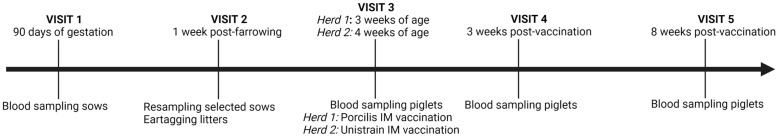
Schematic overview of the study design. Figure was made using Biorender.com.

Two Belgian farrow-to-finish herds were selected based on the presence of routinely PRRSv-vaccinated but ELISA-seronegative sows as determined in a previous field study [15]. Herd 1 had 400 sows on site which were routinely intramuscularly PRRSv-vaccinated with the Porcilis^®^ vaccine (MSD Animal Health, Rahway, NJ, USA). Herd 2 had 300 sows on site which were routinely intramuscularly PRRSv-vaccinated with the Unistrain^®^ vaccine (HIPRA, Amer, Spain). Both herds used the same PRRSv vaccination schedule: one vaccination at 60 days of gestation and one vaccination at 6 days post-farrowing (60–6 schedule). Herd 1 practiced a PCV2 blanket vaccination of the sows (every 3 months) and PCV2 vaccination of the piglets at 28 days of age. Herd 2 only practiced PCV2 vaccination of the breeding gilts (at 35 days of age). Both herds were visited five times (Figure 1). During the first visit, 50 sows in herd 1 and 40 sows in herd 2 were blood sampled at 90 days of gestation. Based on the PRRSv serostatus of the sampled sows, eight sows were selected in each herd for follow-up of their progeny. During the second visit (1 week post-farrowing), selected sows were resampled to confirm their PRRSv serostatus, and the litters of the selected sows were ear-tagged for follow-up. In herd 1, 29 piglets born from 3 ELISA-seropositive sows (E+ piglets) and 54 piglets born from 5 ELISA-seronegative sows (E− piglets) were completely followed throughout the study. In herd 2, 59 piglets born from 5 ELISA-seropositive sows (E+ piglets) and 31 piglets born from 3 ELISA-seronegative sows (E− piglets) were completely followed. The third visit took place at 3 weeks post-farrowing in herd 1 and at 4 weeks post-farrowing in herd 2. During this visit, all piglets were blood sampled, and approximately two-thirds of each selected litter was intramuscularly PRRSv-vaccinated with either Porcilis^®^ (herd 1) or Unistrain^®^ (herd 2). The time of vaccination (herd 1: 3 weeks of age; herd 2: 4 weeks of age) was based on the vaccine leaflet. The remaining third of each selected litter remained unvaccinated and acted as controls throughout the study (Table 1). The control and vaccinated piglets were housed in separate pens (but in the same compartment) post-weaning to avoid horizontal transfer of the vaccine strain to the control piglets. The fourth and fifth visits took place at 3 and 8 weeks post-vaccination (wpv), respectively, and consisted of a blood sampling of the vaccinated piglets. During these visits, the control piglets were resampled as well (herd 1 at 6 weeks and 11 weeks of age; herd 2 at 7 weeks and 12 weeks of age).

### 2.2. Blood Sampling

All blood samples were taken via puncture of the vena jugularis externa. Blood was collected in Vacutainer^®^ SST II Advance tubes (Becton Dickinson, Franklin Lakes, NJ, USA). Blood tubes were cold-transported on the same day to the laboratories of Sciensano (Brussels, Belgium). On arrival, the tubes were centrifuged (1000 g-15 min−4 °C), and serum was stored at −20 °C until analysis.

### 2.3. Enzyme-Linked Immunosorbent Assay (ELISA)

The presence of PRRSv-specific antibodies (Abs) was assessed by means of two commercially available ELISA kits. The IDEXX PRRS X3 Ab test (ELISA 1) (IDEXX Laboratories, Westbrook, ME, USA) was used to analyze the presence of PRRSv-specific Abs directed against recombinant PRRSv-1 and PRRSv-2 ORF7 antigens and is considered the gold standard for PRRSv Ab testing [4]. Additionally, Abs directed against a specific PRRSv-1 antigen-gylcoprotein-rich extract were analyzed using the CIVTEST SUIS PRRS E/S test (ELISA 2) (HIPRA, Amer, Spain) [18]. The presence of porcine circovirus type 2 (PCV2)-specific Abs was assessed using the Biochek PCV2 Antibody Test (Biochek, Reeuwijk, The Netherlands). All ELISA tests were performed according to the manufacturers’ guidelines, without modifications. All washing steps were performed using the WellWash Versa Microplate Washer (Thermo Fisher Scientific, Waltham, MA, USA). Absorbance at 650 nm (ELISA 1), 450 nm (ELISA 2) and 405 nm (PCV2 ELISA) were measured using the MultiSkan FC MicroPlate Photometer (Thermo Fisher Scientific, Waltham, MA, USA). Samples with a sample-to-positive (S/*p*) ratio ≥ 0.4 or ≥ 0.5 were considered to be seropositive in ELISA 1 or the PCV2 ELISA, respectively. Samples with a relative index percent (IRPC value) > 20 were considered to be seropositive in ELISA 2. 

### 2.4. Virus Neutralization Assay (VN)

The presence of PRRSv-specific neutralizing antibodies (NAbs) was analyzed using a virus neutralization assay (VN), as previously described [15,19]. For all samples originating from herd 1, VN was performed against 100 TCID50 of the PRRSv-1 DV strain (the strain from which the Porcilis^®^ vaccine is derived). For all samples originating from herd 2, VN was performed against 100 TCID50 of the PRRSv-1 VP-046 strain (the strain from which the Unistrain^®^ vaccine is derived). Samples with a VN titer ≥ 2 Log_2_ were considered to be seropositive in VN [20].

### 2.5. RNA Extraction and Real-Time Polymerase Chain Reaction (qPCR) 

The IndiMag Pathogen kit and IndiMag 48s instrument (Indical Bioscience, Leipzig, Germany) were used to extract RNA from each serum sample. The presence of PRRSv-1 and PRRSv-2 RNA in the extracted RNA was analyzed via qPCR using the VetMAX PRRSv EU and NA 2.0 kit (Thermo Fisher Scientific, Waltham, MA, USA) on the QuantStudio 5 Real-Time PCR System (Thermo Fisher Scientific, Waltham, MA, USA). Both RNA extraction and qPCR were performed according to the manufacturers’ guidelines.

### 2.6. Statistical Analysis

GraphPad Prism 9 (GraphPad Software, San Diego, CA, USA) was used for visualization and statistical analysis of the results. To compare the presence of PRRSv-specific or PCV2-specific MDAs between E+ piglets and E− piglets, a two-step analysis was performed. Firstly, an MDA ratio was calculated for each sow by dividing the number of PRRSv-seropositive or PCV2-seropositive piglets, at first sampling, by the total number of piglets originating from the sow. Secondly, an unpaired *t*-test was performed to compare the mean MDA-ratios of the E+ sows and E− sows in each herd. A similar two-step analysis was performed to compare the PRRSv-vaccine responses between the E+ and E− piglets. Firstly, a response ratio was calculated for each sow by dividing the number of PRRSv-vaccinated, responding ELISA, VN or PCR piglets by the total number of PRRSv-vaccinated piglets originating from the sow. Secondly, an unpaired t-test was performed to compare the mean response ratios of the E+ sows and E− sows in each herd. These two-step analyses were performed to counteract the possible clustering effect of the sows. Differences in mean ELISA 1 S/*p* values, ELISA 2 IRPC values, PCV2 S/*p* values or VN titers were calculated using an unpaired t-test. Finally, the mean ELISA 1 S/*p* values, ELISA 2 IRPC values, PCV2 S/*p* values, VN titers and PCR Ct-values were written as mean ± standard deviation. All *p*-values > 0.05 were considered to be significant.

## 3. Results

### 3.1. Sow Selection

At 90 days of gestation, 1 month after the last routine PRRSv-vaccination, 50 and 40 breeding sows were blood-sampled in herd 1 and herd 2, respectively. All sampled sows were tested using ELISA 1 and ELISA 2 to determine their PRRSv serostatus (Supplementary Figure S1). In herd 1, 4/50 (8%) sows tested seronegative in ELISA 1, with 3 of these sows testing seronegative in ELISA 2 as well. The remaining ELISA 1 seronegative sow was considered to be low seropositive in ELISA 2, with an IRPC value equal to 28.04. Additionally, one sow tested (low) seropositive in ELISA 1 (S/*p* = 0.55), but seronegative in ELISA 2. In herd 2, 2/40 (5%) sows tested seronegative in both ELISA 1 and ELISA 2. Three additional sows tested seronegative in ELISA 2, but were seropositive in ELISA 1, with S/*p* values equal to 0.43, 0.48 and 0.82. 

Based on this first sampling, eight sows were selected in each herd. The selected sows were resampled at one week post-farrowing to determine their PRRSv serostatus post-farrowing. Sows testing seronegative in either or both ELISA kits were classified as being PRRSv-seronegative. Sows testing seropositive in both ELISA kits were considered to be PRRSv-seropositive (Table 2). One exception was made for sow 4 of herd 1; this sow was considered to be PRRSv-seronegative, despite testing seropositive in both ELISA kits. The reason for the seronegative classification was the fact that the sow had very low ELISA values (S/*p* value ELISA 1 = 0.63 and IRPC value ELISA 2 = 20.1), close to the cut-off values for seropositivity. Additionally, the sow had a complete absence of NAbs (VN titer = 0 Log_2_).

### 3.2. Presence of Maternally-Derived Antibodies

#### 3.2.1. Presence of PRRSv-Specific MDAs

**Figure 2:**
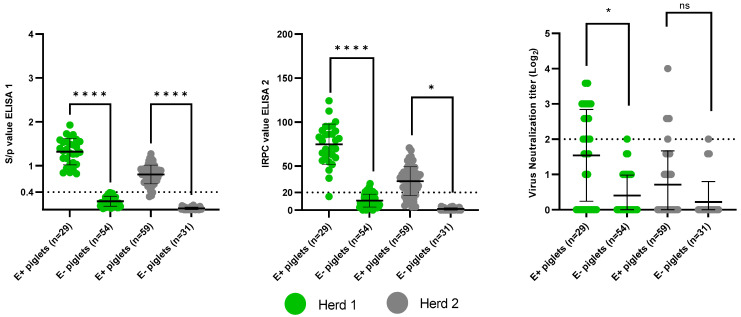
Presence of PRRSv-specific maternally-derived antibodies (MDAs) in piglets born from PRRSv-vaccinated seropositive sows (E+ piglets) and PRRSv-vaccinated seronegative sows (E− piglets) in two Belgian farrow-to-finish herds. PRRSv-specific MDAs were analyzed at 3 weeks of age (herd 1) or 4 weeks of age (herd 2). MDAs were analyzed using IDEXX ELISA (ELISA 1–**left**), CIVTEST ELISA (ELISA 2–**middle**) and virus neutralization assay (VN–**right**). Individual ELISA 1 S/*p* values, ELISA 2 IRPC values or VN titers (Log_2_) are shown as dots on the graphs. Cut-off values for seropositivity are indicated with a dotted line. An unpaired t-test was used to compare the MDA ratios between E+ sows and E− sows. **** *p*-value < 0.0001; * *p*-value < 0.05; ns = not significant.

The presence of PRRSv-specific MDAs at 3 weeks of age (herd 1) or 4 weeks of age (herd 2) was analyzed using ELISA 1, ELISA 2 and VN (Figure 2). A trend was observed between the sow serostatus and the piglet serostatus, with the higher the sow ELISA 1 S/*p* value, ELISA 2 IRPC value and VN titer, the higher the mean ELISA 1 S/*p* values, ELISA 2 IRPC values and VN titers of the respective piglets (Table 3).

The E+ piglets had a clear presence of ELISA 1 MDAs; 29/29 (100%) and 56/59 (94.9%) of the E+ piglets tested seropositive in herd 1 and herd 2, respectively. In contrast, there was an absolute absence of ELISA 1 MDAs in the E− piglets; 0/54 and 0/31 of the E− piglets tested seropositive in herd 1 and herd 2, respectively. ELISA 2 MDAs were present in 28/29 (96.6%) and 45/59 (76.3%) of the E+ piglets in herd 1 and herd 2, respectively. In herd 1, 6/54 (11.1%) of the E− piglets tested seropositive in ELISA 2, while all 31 E− piglets of herd 2 tested seronegative. The six ELISA 2 seropositive E− piglets of herd 1 had low IRPC values, ranging from 20.99 to 30.05. In herd 1, 14/29 (48.3%) E+ piglets tested seropositive in VN, while only 1/54 (1.9%) E− piglets tested seropositive in VN. The VN titer of the one seropositive E− piglet was equal to 2 Log_2_ (the cut-off value for seropositivity). In herd 2, 7/59 (11.9%) E+ piglets were seropositive in VN, compared to only 1/31 (3.2%) E− piglets. The VN titer of the one seropositive E− piglet was equal to 2 Log_2_.

Individual ELISA 1, ELISA 2 and VN MDA ratios for each sow can be found in Supplementary Table S1. In herd 1, the mean ELISA MDA ratio of the E+ sows (ELISA 1: 1.0 ± 0.0; ELISA 2: 0.96 ± 0.06) was significantly higher (*p* < 0.0001) than the mean ELISA MDA ratio of the E− sows (ELISA 1: 0.0 ± 0.0; ELISA 2: 0.1 ± 0.13). Additionally, the mean VN MDA ratio of the E+ sows (0.49 ± 0.43) was significantly higher (*p* = 0.042) than the mean VN MDA ratio of the E− sows (0.018 ± 0.041). In herd 2, the mean ELISA 1 MDA ratio of the E+ sows (0.95 ± 0.12) was significantly higher (*p* < 0.0001) than the mean ELISA 1 MDA ratio of the E− sows (0.0 ± 0.0). The mean ELISA 2 MDA ratio of the E+ sows (0.77 ± 0.44) was also considered significantly higher (*p* = 0.025) than the mean ELISA 2 MDA ratio of the E− sows (0.0 ± 0.0). Finally, no significant difference (*p* = 0.64) was observed between the VN MDA ratio of the E+ sows (0.13 ± 0.28) and the VN MDA ratio of the E− sows (0.04 ± 0.07).

#### 3.2.2. Presence of PCV2-Specific MDAs: Control for Colostrum Intake

All selected sows (both PRRSv-seropositive and PRRSv-seronegative) tested seropositive in PCV2 ELISA (Supplementary Figure S2a). Additionally, the mean PCV2 S/*p* values of the PRRSv-seropositive sows in herd 1 (2.20 ± 0.10) and herd 2 (1.47 ± 0.68) did not differ from the mean PCV2 S/*p* values of the PRRSv-seronegative sows in herd 1 (2.17 ± 0.14) and herd 2 (1.46 ± 0.50). This allowed us to use the presence of PCV2-specific MDAs as a control for adequate colostrum intake of the selected piglets (Supplementary Figure S2b).

In herd 1, all E+ and all E− piglets tested seropositive for the presence of PCV2 MDAs. Furthermore, the mean PCV2 S/*p* value of the E+ piglets (1.91 ± 0.11) did not differ from the mean PCV2 S/*p* value of the E− piglets (1.86 ± 0.15). In herd 2, 32/59 (54.2%) of the E+ piglets and 15/31 (48.4%) of the E− piglets tested PCV2-seropositive. Additionally, there was no significant difference (*p* = 0.73) between the PCV2 MDA ratio of the E+ sows (0.58 ± 0.53) and the PCV2 MDA ratio of the E− sows (0.45 ± 0.43). Finally, no difference was observed between the mean PCV2 S/*p* value of the E+ piglets (0.80 ± 0.53) and the mean PCV2 S/*p* value of the E− piglets (0.68 ± 0.46).

### 3.3. Antibody Responses Post-Vaccination

#### 3.3.1. Antibody Responses 3 Weeks Post-Vaccination

The presence of an antibody response at 3 weeks post-vaccination (wpv) was defined by either seroconversion (in the case of piglets testing seronegative pre-vaccination) or by an increased or unchanged ELISA 1 S/*p* value, ELISA 2 IRPC value or VN titer (in the case of piglets testing seropositive pre-vaccination). An absence of antibody response at 3 weeks post-vaccination was defined by no seroconversion (in the case of piglets testing seronegative pre-vaccination) or by a decreased ELISA 1 S/*p* value, ELISA 2 IRPC value or VN titer (in the case of piglets testing seropositive pre-vaccination, which is due to waning MDAs). 

**Figure 3:**
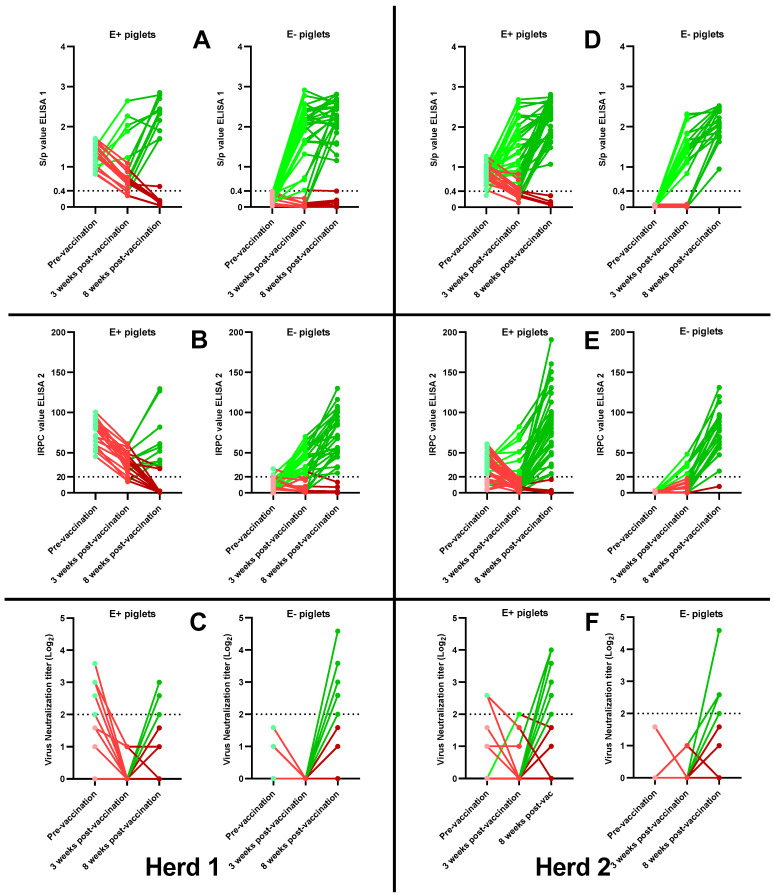
PRRSv-antibody responses in PRRSv-vaccinated piglets born from PRRSv-vaccinated seropositive sows (E+ piglets) and PRRSv-vaccinated seronegative sows (E− piglets) in two Belgian farrow-to-finish herds. Piglets in herd 1 (**A**–**C**) were intramuscularly PRRSv-vaccinated at 3 weeks of age with the Porcilis^®^ vaccine. Piglets in herd 2 (**D**–**F**) were intramuscularly PRRSv-vaccinated at 4 weeks of age with the Unistrain^®^ vaccine. Antibody responses at 3 and 8 weeks post-vaccination were analyzed using IDEXX ELISA (A + D; ELISA 1), CIVTEST ELISA (B + E; ELISA 2) and virus neutralization assay (C + F; VN). Individual S/*p* values (ELISA 1), IRPC values (ELISA 2) and Log_2_ VN titers are shown as dots with lines connecting the values pre-vaccination, 3 weeks post-vaccination and 8 weeks post-vaccination for each piglet. Light green lines indicate the presence of an antibody response at 3 weeks post-vaccination, while dark green lines indicate the presence of an antibody response by 8 weeks post-vaccination. Light red lines indicate the absence of an antibody response at 3 weeks post-vaccination, while dark red lines indicate the absence of an antibody response by 8 weeks post-vaccination. Cut-off values for seropositivity are included in each graph as a dotted horizontal line.

In herd 1, 26/36 (72.2%) E− piglets and 5/20 (25%) E+ piglets showed an ELISA 1 antibody response at 3 wpv (Figure 3A). The ELISA 1 response ratio of the E− sows (0.72 ± 0.27) was significantly higher (*p* = 0.036) than the ELISA 1 response ratio of the E+ sows (0.22 ± 0.22). In ELISA 2, 20/36 (55.6%) E− piglets and 0/20 (0%) E+ piglets showed an antibody response at 3 wpv (Figure 3B). The ELISA 2 response ratio of the E− sows (0.59 ± 0.28) was significantly higher (*p* = 0.013) than the ELISA 2 response ratio of the E+ sows (0.0 ± 0.0). Finally, 0/36 (0%) E− piglets and 0/20 (0%) E+ piglets had a neutralizing antibody response at 3 wpv (Figure 3C). 

In herd 2, 16/20 (80%) E− piglets and 24/39 (61.5%) E+ piglets showed an ELISA 1 antibody response at 3 wpv (Figure 3D). The ELISA 1 response ratio of the E− sows (0.79 ± 0.20) was not significantly higher (*p* = 0.45) than the ELISA 1 response ratio of the E+ sows (0.60 ± 0.37). In ELISA 2, 6/20 (30%) E− piglets and 9/39 (23.1%) E+ piglets had an antibody response at 3 wpv (Figure 3E). The ELISA 2 response ratio of the E− sows (0.31 ± 0.17) was not significantly higher (*p* = 0.68) than the ELISA 2 response-ratio of the E+ sows (0.22 ± 0.31). Finally, 0/20 (0%) E− piglets and 1/39 (2.6%) E+ piglets had a neutralizing antibody response at 3 wpv (Figure 3F). 

The ELISA 1, ELISA 2 and VN response ratios for each sow can be found in Supplementary Table S2.

#### 3.3.2. Antibody Responses 8 Weeks Post-Vaccination

The presence of an antibody response by 8 wpv was defined by either seroconversion (in the case of piglets testing seronegative at 3 wpv) or by a further increase in ELISA 1 S/*p* value, ELISA 2 IRPC value or VN titer. Additionally, piglets that showed an antibody response at 3 wpv and remained seropositive at the end of the study were considered to have an antibody response by 8 wpv. An absence of antibody response by 8 wpv was defined for piglets who remained seronegative throughout the study, piglets who became seronegative in the period between 3 wpv and 8 wpv and seropositive piglets who had a declining ELISA 1 S/*p* value, ELISA 2 IRPC value or VN titer at 3 wpv which further declined by 8 wpv. 

In herd 1, 28/36 (77.8%) E− piglets and 11/20 (55%) E+ piglets showed an ELISA 1 antibody response by the end of the study (Figure 3A). The ELISA 1 response ratio of the E− sows (0.77 ± 0.20) was not significantly higher (*p* = 0.50) than the ELISA 1 response ratio of the E+ sows (0.59 ± 0.53). The ELISA 1 S/*p* values of the 28 responding E− piglets ranged from 1.16 to 2.81, with a mean S/*p* value of 2.25 ± 0.44. The ELISA 1 S/*p* values of the 11 responding E+ piglets ranged from 1.70 to 2.85, with a mean S/*p* value of 2.31 ± 0.40. In ELISA 2, 27/36 (75%) E− piglets and 9/20 (45%) E+ piglets had an antibody response by 8 wpv (Figure 3B). The ELISA 2 response ratio of the E− sows (0.75 ± 0.14) was not significantly higher (*p* = 0.18) than the ELISA 2 response ratio of the E+ sows (0.45 ± 0.41). The ELISA 2 IRPC values of the 27 responding E− piglets ranged from 24.71 to 130.13, with a mean IRPC value of 73.17 ± 28.35. The ELISA 2 IRPC values of the 9 responding E− piglets ranged from 35.36 to 129.56, with a mean IRPC value of 69.40 ± 36.11. In VN, 16/36 (44.4%) E− piglets and 3/20 (15%) E+ piglets had a neutralizing antibody response by the end of the study (Figure 3C). The VN response ratio of the E− sows (0.48 ± 0.24) was not considered significantly higher (*p* = 0.057) than the VN response ratio of the E+ sows (0.13 ± 0.12). VN titers of the 16 responding E− piglets ranged from 2 Log_2_ to 4.585 Log_2_, with a mean VN titer of 2.66 ± 0.77 Log_2_. VN titers of the 3 responding E+ piglets were equal to 2 Log_2_, 2.585 Log_2_ and 3 Log_2_.

In herd 2, 20/20 (100%) of the E− piglets and 35/39 (89.7%) of the E+ piglets showed an ELISA 1 antibody response by 8 wpv (Figure 3D). The ELISA 1 response ratio of the E− sows (1.0 ± 0.0) was not significantly higher (*p* = 0.28) than the ELISA 1 response ratio of the E+ sows (0.90 ± 0.14). The ELISA 1 S/*p* values of the 20 responding E− piglets ranged from 0.95 to 2.52, with a mean S/*p* value of 2.09 ± 0.40. The ELISA 1 S/*p* values of the 35 responding E+ piglets ranged from 1.07 to 2.81, with a mean S/*p* value of 2.22 ± 0.44. In ELISA 2, 19/20 (95%) E− piglets and 35/39 (89.7%) E+ piglets had an antibody response at 8 wpv (Figure 3E). The ELISA 2 response ratio of the E− sows (0.93 ± 0.12) was not significantly higher (*p* = 0.65) than the ELISA 2 response ratio of the E+ sows (0.90 ± 0.095). The ELISA 2 IRPC values of the 19 responding E− piglets ranged from 27.25 to 131.09, with a mean IRPC value of 80.11 ± 26.00. The ELISA 2 IRPC values of the 35 responding E+ piglets ranged from 23.35 to 190.69, with a mean IRPC value of 86.65 ± 41.21. Finally, a neutralizing antibody response was observed in 8/20 (40%) E− piglets and in 9/39 (23.1%) E+ piglets (Figure 3F). The VN response ratio of the E− sows (0.40 ± 0.24) was not significantly higher (*p* = 0.27) than the VN response ratio of the E+ sows (0.23 ± 0.16). VN titers of the 8 responding E− piglets ranged from 2 Log_2_ to 4.58 Log_2_, with a mean VN titer of 2.62 ± 0.85 Log_2_. VN titers of the 9 responding E+ piglets ranged from 2 Log_2_ to 4 Log_2_, with a mean VN titer of 3.02 ± 0.90 Log_2_.

The ELISA 1, ELISA 2 and VN response ratios for each sow can be found in Supplementary Table S3.

#### 3.3.3. Correlation between MDAs and the Absence of an Antibody Response Post-Vaccination

Since there was a lack of an antibody response in a substantial proportion of the E+ and E− piglets in herd 1, a secondary analysis was performed to have a better understanding of the relationship between the MDAs pre-vaccination and the absence of antibody responses post-vaccination. For this analysis, the mean ELISA 1 S/*p* values, ELISA 2 IRPC values and VN titers pre-vaccination were compared between the piglets of herd 1 with and without an ELISA 1 antibody response at the end of the study.

There was a high proportion of E+ piglets (9/20; 45%) that did not have an ELISA 1 antibody response by 8 wpv. Before vaccination, the mean ELISA 1 S/*p* value (1.32 ± 0.29) and ELISA 2 IRPC value (74.70 ± 19.86) of the 9 E+ piglets without an ELISA 1 antibody response did not significantly differ from the mean ELISA 1 S/*p* value (1.23 ± 0.29) and ELISA 2 IRPC value (79.47 ± 13.84) of the 11 E+ piglets with an ELISA 1 antibody response. The mean, pre-vaccination VN titer (1.97 ± 1.33) of the 9 E+ piglets without an ELISA 1 antibody response was higher than the mean, pre-vaccination VN titer (0.98 ± 1.32) of the 11 E+ piglets with an ELISA 1 antibody response, but this difference was not considered significant (*p* = 0.11). 

Interestingly, there was also a high proportion of E− piglets that lacked an ELISA 1 antibody response by the end of the study (8/36; 22.2%). Since none of the vaccinated E− piglets of herd 1 showed the presence of MDAs (with the exception of two piglets that were very low seropositive in ELISA 2), this secondary analysis could not be performed, and the absence of an ELISA 1 antibody response in the E− piglets could not be explained by a difference in the presence of MDAs.

### 3.4. Vaccine Viremia Post-Vaccination

#### 3.4.1. Vaccine Viremia 3 Weeks Post-Vaccination

**Figure 4:**
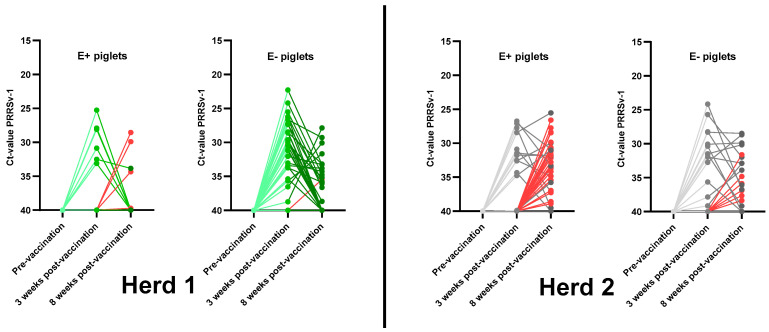
PRRSv vaccine viremia in PRRSv-vaccinated piglets born from PRRSv-vaccinated seropositive sows (E+ piglets) and PRRSv-vaccinated seronegative sows (E− piglets) in two Belgian farrow-to-finish herds. Piglets in herd 1 (**left**) were intramuscularly PRRSv-vaccinated at 3 weeks of age with the Porcilis^®^ vaccine. Piglets in herd 2 (**right**) were intramuscularly PRRSv-vaccinated at 4 weeks of age with the Unistrain^®^ vaccine. Results are shown as dots for the individual Ct-values at pre-vaccination and at 3 weeks and 8 weeks post-vaccination, with lines connecting the Ct-values for each piglet over time. Red lines indicate a secondary vaccine exposure—piglets that became PCR-positive between 3 weeks and 8 weeks post-vaccination. Piglets testing negative in PCR were given a Ct-value of 40.

In herd 1 (Figure 4, left), 27/36 (75%) E− piglets and 6/20 (30%) E+ piglets tested PCR-positive at 3 wpv. The PCR response ratio of the E− sows (0.74 ± 0.25) was significantly higher (*p* = 0.042) than the PCR response ratio of the E+ sows (0.25 ± 0.28). The Ct-values of the 27 PCR-positive E− piglets ranged from 22.29 to 38.75, with a mean Ct-value of 30.21 ± 3.96. The Ct-values of the 6 PCR-positive E+ piglets ranged from 25.27 to 33.12, with a mean Ct-value of 29.62 ± 3.05. 

In herd 2 (Figure 4, right), 13/20 (65%) E− piglets and 12/39 (31%) E+ piglets tested PCR positive at 3 wpv. The PCR response ratio of the E− sows (0.63 ± 0.14) was significantly higher (*p* = 0.048) than the PCR response ratio of the E+ sows (0.31 ± 0.19). The Ct-values of the 13 PCR-positive E− piglets ranged from 24.15 to 39.16, with a mean Ct-value of 31.32 ± 4.37. The Ct-values of the 12 PCR-positive E+ piglets ranged from 26.74 to 39.91, with a mean Ct-value of 31.52 ± 3.79.

The PCR response ratio for each sow can be found in Supplementary Table S2.

#### 3.4.2. Vaccine Viremia 8 Weeks Post-Vaccination

In herd 1 (Figure 4, left) 1, 17/36 (46%) E− piglets and 5/20 (25%) E+ piglets tested PCR-positive at 8 wpv. The PCR response ratio of the E− sows (0.48 ± 0.22) was not significantly higher (*p* = 0.30) than the PCR response ratio of the E+ sows (0.27 ± 0.30). Sixteen out of seventeen PCR-positive E− piglets were already PCR-positive at 3 wpv, while one E− piglet became PCR positive between 3 wpv and 8 wpv (secondary vaccine exposure). The Ct-values of the 17 PCR-positive E− piglets ranged from 27.86 to 38.68, with a mean Ct-value of 33.83 ± 2.80. In contrast, only one out of five PCR positive E+ piglets was already PCR-positive at 3 wpv; the remaining four PCR-positive E+ piglets became PCR-positive between 3 wpv and 8 wpv (secondary vaccine exposure). The Ct-values of the 5 PCR positive E+ piglets ranged from 28.55 to 39.72, with a mean Ct-value of 33.27 ± 4.38.

In herd 2 (Figure 4, right), 17/20 (85%) E− piglets and 36/39 (92%) E+ piglets tested PCR positive at 8 wpv. The PCR response ratio of the E− sows (0.84 ± 0.045) was not significantly lower (*p* = 0.48) than the PCR response ratio of the E+ sows (0.91 ± 0.15). Eleven out of seventeen PCR-positive E− piglets were already PCR-positive at 3 wpv, and six E− piglets became PCR-positive between 3 wpv and 8 wpv (secondary vaccine exposure). The Ct-values of the 17 PCR-positive E− piglets ranged from 28.48 to 39.17, with a mean Ct-value of 33.88 ± 3.41. Eleven out of thirty-six PCR-positive E+ piglets were already PCR-positive at 3 wpv; an additional twenty-five E+ piglets became PCR-positive between 3 wpv and 8 wpv (secondary vaccine exposure). The Ct-values of the 36 PCR-positive E+ piglets ranged from 25.54 to 39.51, with a mean Ct-value of 32.79 ± 3.40.

The PCR response ratio for each sow can be found in Supplementary Table S3.

### 3.5. Absence of Field PRRSv Circulation: Waning of PRRSv-Specific MDAs in Control Piglets

**Figure 5:**
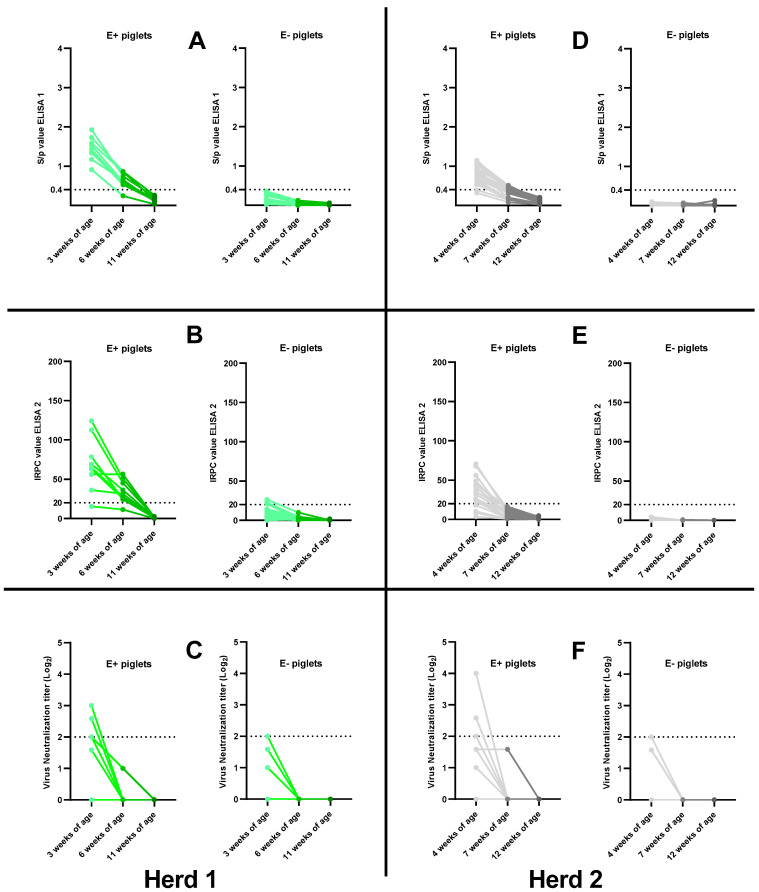
Serological follow-up of PRRSv-specific antibodies in unvaccinated, control piglets born from PRRSv-vaccinated seropositive sows (E+ piglets) and PRRSv-vaccinated seronegative sows (E− piglets) in two Belgian farrow-to-finish herds. Piglets in herd 1 (**A**–**C**) were sampled at 3, 6 and 11 weeks of age. Piglets in herd 2 (**D**–**F**) were sampled at 4, 7 and 12 weeks of age. All piglets were analyzed using IDEXX ELISA (A + D; ELISA 1), CIVTEST ELISA (B + E; ELISA 2) and virus neutralization assay (C + F; VN). Individual S/*p* values (ELISA 1), IRPC values (ELISA 2) and Log_2_ VN titers are shown as dots with lines connecting the values at each sample moment for each piglet. Cut-off values for seropositivity are included in each graph as a dotted horizontal line.

To ensure the absence of field PRRSv circulation in the selected herds, control piglets (unvaccinated) were included in the study. All control piglets remained PRRSv-1- and PRRSv-2-PCR negative throughout the study. Waning of PRRSv-specific MDAs was observed in control piglets testing seropositive at first sampling. Control piglets testing seronegative at first sampling remained seronegative throughout the study (Figure 5).

In herd 1, one E+ control piglet became ELISA 1-seronegative by 6 weeks of age (woa), while the remaining eight E+ control piglets became ELISA 1-seronegative between 6 woa and 11 woa (Figure 5A). In ELISA 2, one E+ control piglet was already seronegative at 3 woa, and the remaining eight E+ control piglets became seronegative in the period between 6 woa and 11 woa (Figure 5B). Six E+ control piglets were seropositive in VN at 3 woa, but these piglets became seronegative in the period between 3 woa and 6 woa (Figure 5C). The eighteen E− control piglets of herd 1 remained seronegative in ELISA 1 throughout the study (Figure 5A). At 3 woa, four E− control piglets were seropositive in ELISA 2, with IRPC values close to the cut-off value, but these all became seronegative in the period between 3 woa and 6 woa (Figure 5B). Finally, one E− control piglet was considered VN-seropositive at 3 woa, but this piglet tested VN-seronegative at 6 woa (Figure 5C).

In herd 2, two E+ control piglets were already ELISA 1-seronegative at 4 woa, ten E+ control piglets tested ELISA 1-seronegative by 7 woa and eventually all E+ piglets became ELISA 1-seronegative by the end of the study (Figure 5B). Sixteen E+ control piglets were ELISA 2-seropositive at 4 woa, but these all became ELISA 2-seronegative in the period between 4 woa and 7 woa (Figure 5D). Three E+ control piglets were considered VN-seropositive at 4 woa, but these piglets became VN-seronegative by 7 woa (Figure 5F). All E− control piglets remained seronegative in both ELISA 1 and ELISA 2 throughout the study (Figure 5D,E). One E− control piglet tested seropositive in VN at 4 woa, but this piglet became seronegative by 7 woa (Figure 5F). 

## 4. Discussion

Vaccination against PRRSv is widely practiced in sows and/or piglets. Veterinary field reports stating the presence of routinely PRRSv-vaccinated, but ELISA-seronegative, sows formed the basis for the first cross-sectional prevalence study concerning this phenomenon [15]. Results of PCV2 ELISA in the current study showed that the PRRSv-vaccinated, but ELISA-seronegative, sows do not have a B-cell deficiency, since they have a clear presence of PCV2 Abs. This suggests that the observed phenomenon of seronegativity despite routine vaccination is a PRRSv-specific issue. However, the specific reasons why these PRRSv-vaccinated sows test ELISA-seronegative remain unknown and warrant further investigation. In the current study, the possible consequences of the PRRSv-seronegative sow status (despite routine vaccination) for the progeny were investigated in two Belgian farrow-to-finish herds. 

As expected, a clear difference in the presence of PRRSv-specific MDAs was observed between piglets born from PRRSv-vaccinated ELISA-seropositive sows (E+ piglets) and piglets born from PRRSv-vaccinated ELISA-seronegative sows (E− piglets) both at 3 weeks of age (herd 1) and 4 weeks of age (herd 2). Most E+ piglets included in this study received MDAs specific for PRRSv through colostrum intake. In contrast, the E− piglets did not receive PRRSv-specific MDAs, despite adequate colostrum intake (presence of PCV2-specific MDAs). Additionally, about half of the E+ piglets tested VN-seropositive in herd 1 and thus received MDNAs. At 4 weeks of age (herd 2), only 12% of the E+ piglets had MDNAs. In both herds, an absence of MDNAs was found in the E− piglets. 

The observed differences in PRRSv-specific MDAs had an apparent effect on the PRRSv vaccine responses. In herd 1, both the early (3 wpv) antibody response and vaccine viremia were significantly higher in the E− piglets compared to the E+ piglets. It is quite evident that the presence of both MDAs and MDNAs in the E+ piglets causes these piglets to lack an early PRRSv vaccine response, which is consistent with other studies [16,17]. In herd 2, the difference in early vaccine responses was less pronounced, with both the E− piglets and E+ piglets having a strong early antibody response. Since the piglets in herd 2 were vaccinated one week later than the piglets in herd 1, waning of a large part of the MDAs and MDNAs had already took place prior to vaccination. However, there was still a significant difference in early vaccine viremia between the E− and E+ piglets of herd 2, despite the later vaccination. Further research is needed to understand why the E+ piglets in herd 2 showed such a low initial vaccine viremia despite the relatively low presence of MDAs at the moment of vaccination.

In herd 1, there was a high proportion of piglets (both E− piglets and E+ piglets) that tested ELISA-seronegative by 8 wpv and thus did not respond to the PRRSv vaccination. The lack of a vaccine responses in the E+ piglets can be explained by the high amount of MDAs at the moment of vaccination, which interfered with antibody responses [16,17]. However, MDAs were absent in the E− piglets at the moment of vaccination. It could be hypothesized that there is some genetic factor causing the E− piglets not to respond to the PRRSv vaccination (as with their respective mother sows). Alternatively, there could be some transfer of cell-mediated immunity from sow to piglet via colostrum, which interferes with the PRRSv vaccination response. Finally, it is noteworthy that the overall disease status in the herds was not investigated, and it could be hypothesized that a high disease pressure could lead to a reduced immune response to the PRRSv vaccination. Further research is warranted to explore why these E− piglets did not react to PRRSv vaccination. In herd 2, this vaccine non-responsiveness was not observed, with almost all piglets testing ELISA-seropositive by 8 wpv. 

Interestingly, there were some clear differences in the duration of vaccine viremia and the magnitude of secondary vaccine exposure between the vaccinated piglets of herd 1 and herd 2. In short, the vaccine viremia duration and the proportion of piglets with a secondary vaccine exposure was higher in herd 2 compared to herd 1. It can be hypothesized that the time of vaccination is an important driver for this observed difference—less MDAs in herd 2 at the moment of vaccination lead to a longer duration of vaccine viremia and a stronger presence of secondary vaccine exposure. Alternatively, the used vaccine strain might also play a role in the observed difference, since previous studies have shown that the Unistrain^®^ vaccine has a longer-lasting vaccine viremia and a higher transmission rate compared to the Porcilis^®^ vaccine [9,21,22]. In the current study design, no hard statements can be made relating the observed differences in vaccine viremia with the used vaccine strains, since the differences in the herd characteristics between herd 1 and herd 2 might play a major role as well. However, the observed long-lasting vaccine viremia (PCR-positive until at least 8 wpv), and consequently secondary vaccine exposure, raises some further concern about the safety of MLVs, especially in the context of possible recombination events [9,10,11,12,13,14].

In conclusion, the current study provided a first indication of the possible consequences of ELISA-seronegative sow status, despite routine PRRSv-vaccination, for its progeny. The piglets born from these seronegative sows lack the presence of PRRSv-specific MDAs, and due to this, they show a strong initial vaccine viremia and seroconversion. It could be hypothesized that this lack of MDAs could be biologically relevant in the case of an early PRRSv field infection, with these piglets being less protected compared to the piglets born to ELISA-seropositive sows, who are receiving PRRSv-specific MDAs. 

## Figures and Tables

**Table 1:** Overview of the number of vaccinated and control piglets born from PRRSv-vaccinated ELISA-seropositive sows (E+ piglets) and PRRSv-vaccinated ELISA-seronegative sows (E− piglets) in two Belgian farrow-to-finish herds.

	E+ Piglets		E− Piglets	
	Vaccinated	Control	Vaccinated	Control
Herd 1	20	9	36	18
Herd 2	39	20	20	11

**Table 2:** Overview of the selected PRRSv-vaccinated sows in two Belgian farrow-to-finish herds. Selected sows were blood-sampled at 90 days of gestation (1 month after the last PRRSv vaccination) and at 1 week post-farrowing (1 wpf). The sows were analyzed using IDEXX ELISA (ELISA 1), CIVTEST ELISA (ELISA 2) and virus neutralization assay (VN). Each sow was given a PRRSv serostatus based on the ELISA results of the second sampling (1 wpf).

Sow Number (Parity)	S/*p* ELISA 1 (90d Gestation)	IRPC ELISA 2 (90d Gestation)	S/*p* ELISA 1 (1 wpf)	IRPC ELISA 2 (1 wpf)	Log_2_ VN Titer (1 wpf)	PRRSv Serostatus
*Herd 1*						
Sow 1 (7)	0.36	6.62	0.19	5.84	0.00	Seronegative
Sow 2 (4)	0.39	28.04	0.30	15.56	2.00	Seronegative
Sow 3 (7)	0.79	38.88	0.44	17.45	2.00	Seronegative
Sow 4 (5)	0.65	20.98	0.63	20.10	0.00	Seronegative
Sow 5 (1)	0.27	18.07	0.15	19.86	0.00	Seronegative
Sow 6 (6)	2.20	128.93	1.96	100.85	4.58	Seropositive
Sow 7 (5)	2.32	134.10	2.00	123.99	4.58	Seropositive
Sow 8 (1)	1.82	113.93	1.08	94.21	0.00	Seropositive
*Herd 2*						
Sow 1 (5)	0.14	6.41	0.14	5.96	2.00	Seronegative
Sow 2 (3)	0.43	17.67	0.37	13.12	1.58	Seronegative
Sow 3 (3)	0.29	12.06	0.27	7.50	2.58	Seronegative
Sow 4 (4)	1.93	117.40	1.47	65.04	1.58	Seropositive
Sow 5 (3)	1.75	109.76	1.71	89.25	2.00	Seropositive
Sow 6 (3)	1.30	46.18	1.41	54.30	2.58	Seropositive
Sow 7 (1)	0.59	47.30	2.01	120.24	4.58	Seropositive
Sow 8 (1)	2.05	105.60	1.87	85.11	1.58	Seropositive

**Table 3:** Presence of PRRSv-specific maternally-derived antibodies (MDAs) in piglets born from PRRSv-vaccinated seropositive sows and PRRSv-vaccinated seronegative sows in two Belgian farrow-to-finish herds. Sows were analyzed at 1 week post-farrowing (1 wpf) using IDEXX ELISA (ELISA 1), CIVTEST ELISA (ELISA 2) and virus neutralization assay (VN). Piglets were analyzed at 3 weeks of age (herd 1) or 4 weeks of age (herd 2) using ELISA 1, ELISA 2 and VN. Piglet values are shown as mean ± standard deviation.

Sow Number (Parity)	S/*p* ELISA 1 (1 wpf)	IRPC ELISA 2 (1 wpf)	Log_2_ VN Titer (1 wpf)	S/*p* ELISA 1 Piglets	IRPC ELISA 2 Piglets	Log_2_ VN Titer Piglets
*Herd 1*						
Sow 1 (7)	0.19	5.84	0	0.13 ± 0.02	1.81 ± 1.36	0.70 ± 0.61
Sow 2 (4)	0.30	15.56	2	0.13 ± 0.03	12.67 ± 5.60	0.91 ± 0.30
Sow 3 (7)	0.44	17.45	2	0.28 ± 0.08	12.63 ± 9.73	0.20 ± 0.50
Sow 4 (5)	0.63	20.10	0	0.32 ± 0.04	13.95 ± 4.62	0.33 ± 0.73
Sow 5 (1)	0.15	19.86	0	0.06 ± 0.03	9.66 ± 5.68	0.00 ± 0.00
Sow 6 (6)	1.96	100.85	4.58	1.48 ± 0.21	73.33 ± 14.68	2.49 ± 0.77
Sow 7 (5)	2.00	123.99	4.58	1.46 ± 0.26	85.24 ± 30.89	2.08 ± 1.05
Sow 8 (1)	1.08	94.21	0	1.04 ± 0.19	66.62 ± 19.93	0.10 ± 0.32
*Herd 2*						
Sow 1 (5)	0.14	5.96	2	0.01 ± 0.01	0.39 ± 0.52	0.00 ± 0.00
Sow 2 (3)	0.37	13.12	1.58	0.03 ± 0.04	1.07 ± 1.17	0.63 ± 0.87
Sow 3 (3)	0.27	7.50	2.58	0.04 ± 0.03	1.65 ± 1.20	0.00 ± 0.00
Sow 4 (4)	1.47	65.04	1.58	0.93 ± 0.1	37.27 ± 6.46	0.13 ± 0.35
Sow 5 (3)	1.71	89.25	2	0.78 ± 0.11	38.84 ± 13.60	0.00 ± 0.00
Sow 6 (3)	1.41	54.30	2.58	0.54 ± 0.12	10.50 ± 4.40	0.38 ± 0.52
Sow 7 (1)	2.01	120.24	4.58	0.89 ± 0.34	41.89 ± 16.39	1.99 ± 1.00
Sow 8 (1)	1.87	85.11	1.58	0.87 ± 0.21	35.75 ± 16.15	0.68 ± 0.74

## Data Availability

The data presented in this study are available on request from the corresponding author. The data are not publicly available due to privacy reasons.

## Supplementary Materials

The following supporting information can be downloaded at: https://www.mdpi.com/article/10.3390/v15020479/s1. Figure S1: Presence of PRRSv-specific antibodies in multiple PRRSv-vaccinated sows originating from two Belgian farrow-to-finish herds; Figure S2: (a) Presence of porcine circovirus type 2 (PCV2) antibodies in PRRSv-vaccinated ELISA-seropositive sows (E+ sows) and PRRSv-vaccinated ELISA-seronegative sows (E− sows) originating from two Belgian farrow-to-finish herds; (b) Presence of porcine circovirus type 2 (PCV2) maternally-derived antibodies in piglets born from PRRSv-vaccinated ELISA-seropositive sows (E+ piglets) and piglets born from PRRSv-vaccinated ELISA-seronegative sows (E− piglets) originating from two Belgian farrow-to-finish herds; Table S1: Presence of PRRSv-specific maternally-derived antibodies (MDAs) in sixteen PRRSv-vaccinated sows originating from two Belgian farrow-to-finish herds; Table S2: PRRSv vaccine responses in piglets born from sixteen PRRSv-vaccinated sows originating from two Belgian farrow-to-finish herds; Table S3: PRRSv vaccine responses in piglets born from sixteen PRRSv-vaccinated sows originating from two Belgian farrow-to-finish herds.
