# Supplementary material for: Follow-Up of PRRSv-Vaccinated Piglets Born from PRRSv-Vaccinated, ELISA-Seropositive and ELISA-Seronegative Sows
Source: Viruses. 2023 Feb 9;15(2):479. doi: 10.3390/v15020479 (PMC9967088; doi:10.3390/v15020479)
Supplement: Supplementary file 1 [file viruses-15-00479-s001.zip › Supplementary Figures S1 and S2.pdf]

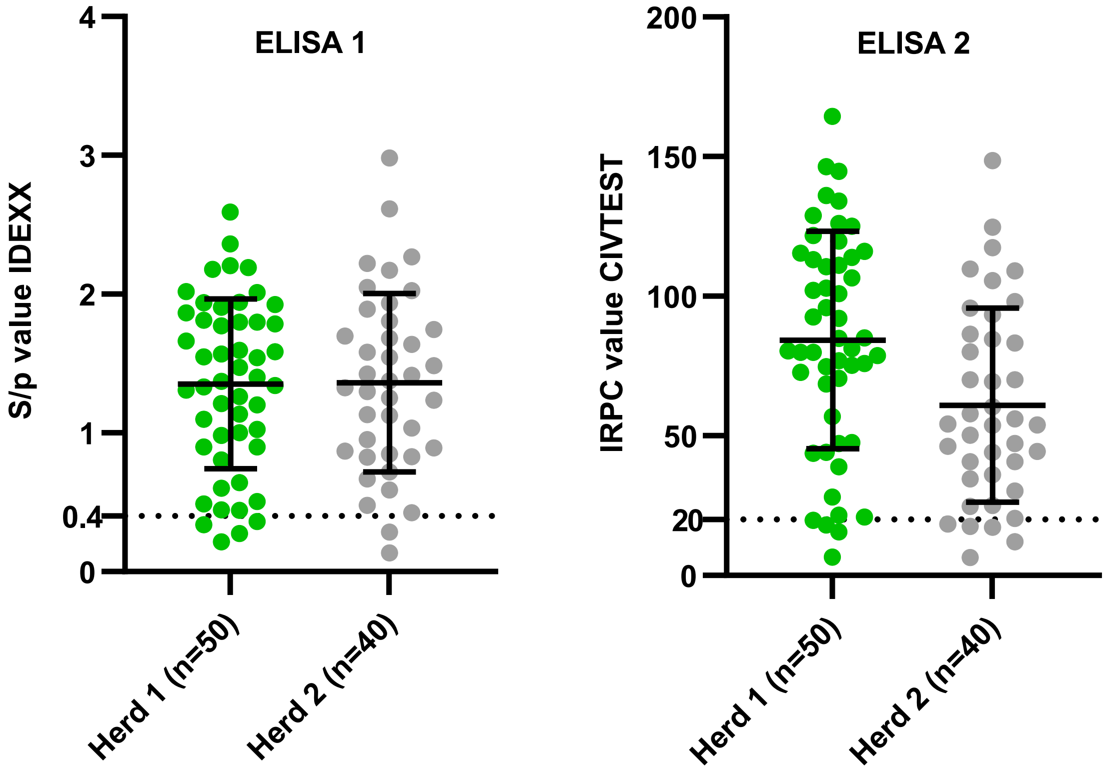

Supplementary Figure S1. Presence of PRRSv-specific antibodies in multiple PRRSv-vaccinated sows originating from two Belgian farrow-to-finish herds. All sows were sampled at 90 days of gestation. Sows of herd 1 were routinely intramuscularly PRRSv-vaccinated with the Porcilis® vaccine at 60 days of gestation and 6 days post-farrowing. Sows of herd 2 were routinely intramuscularly PRRSv-vaccinated with the Unistrain® vaccine at 60 days of gestation and 6 days post-farrowing. IDEXX ELISA (ELISA 1 – left) and CIVTEST ELISA (ELISA 2 – right), were used to analyze the presence of PRRSv-specific antibodies. Results are shown as individual dots for each sow, cut-off value for seropositivity is shown as a dotted line.

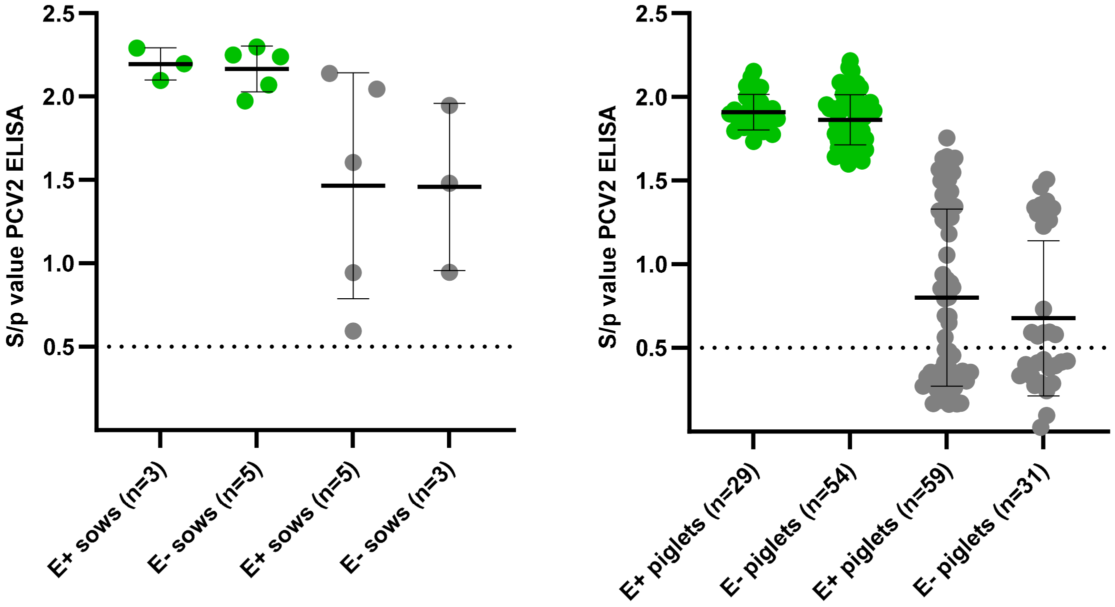

Supplementary Figure S2a (left). Presence of Porcine Circovirus Type 2 (PCV2)-antibodies in PRRSv-vaccinated ELISA seropositive sows (E+ sows) and PRRSv-vaccinated ELISA seronegative sows (E- sows) originating from two Belgian farrow-to-finish herds. PCV2-antibodies were analyzed using the PCV2 Biochek ELISA. Results are shown as individual dots for each sow, cut-off value for seropositivity is shown as a dotted line.

Supplementary Figure S2b (right). Presence of Porcine Circovirus Type 2 (PCV2)-maternally derived antibodies in piglets born from PRRSv-vaccinated ELISA seropositive sows (E+ piglets) and piglets born from PRRSv-vaccinated ELISA seronegative sows (E- piglets) originating from two Belgian farrow-to-finish herds. PCV2-antibodies were analyzed using the PCV2 Biochek ELISA. Results are shown as individual dots for each piglet, cut-off value for seropositivity is shown as a dotted line.
