# Supplementary material for: Follow-Up of PRRSv-Vaccinated Piglets Born from PRRSv-Vaccinated, ELISA-Seropositive and ELISA-Seronegative Sows
Source: Viruses. 2023 Feb 9;15(2):479. doi: 10.3390/v15020479 (PMC9967088; doi:10.3390/v15020479)
Supplement: Supplementary file 1 [file viruses-15-00479-s001.zip › Supplementary Tables.pdf]

**Supplementary Table S1.** Presence of PRRSv-specific maternally-derived antibodies (MDAs) in sixteen PRRSv-vaccinated sows originating from two Belgian farrow-to-finish herds. For each sow the ELISA 1 (IDEXX ELISA), ELISA 2 (CIVTEST ELISA) and VN (virus neutrlization assay) MDA ratio was calculated by dividing the number of PRRSv-seropositive piglets by the total number of piglets originating from each respective sow, at either 3 weeks of age (herd 1) or 4 weeks of age (herd 2).

| Sow number (parity) | MDA ratio ELISA 1 | MDA ratio ELISA 2 | MDA ratio VN |
|---|---|---|---|
| *Herd 1* | | | |
| Sow 1 (7) | 0/8 (0.0) | 0/8 (0.0) | 0/8 (0.0) |
| Sow 2 (4) | 0/11 (0.0) | 1/11 (0.09) | 0/11 (0.0) |
| Sow 3 (7) | 0/13 (0.0) | 4/13 (0.31) | 0/13 (0.0) |
| Sow 4 (5) | 0/11 (0.0) | 1/11 (0.09) | 1/11 (0.09) |
| Sow 5 (1) | 0/11 (0.0) | 0/11 (0.0) | 0/11 (0.0) |
| Sow 6 (6) | 10/10 (1.0) | 10/10 (1.0) | 8/10 (0.8) |
| Sow 7 (5) | 9/9 (1.0) | 8/9 (0.89) | 6/9 (0.67) |
| Sow 8 (1) | 10/10 (1.0) | 10/10 (1.0) | 0/10 (0.0) |
| | | | |
| *Herd 2* | | | |
| Sow 1 (5) | 0/10 (0.0) | 0/10 (0.0) | 0/10 (0.0) |
| Sow 2 (3) | 0/8 (0.0) | 0/8 (0.0) | 1/8 (0.13) |
| Sow 3 (3) | 0/13 (0.0) | 0/13 (0.0) | 0/13 (0.0) |
| Sow 4 (4) | 12/12 (1.0) | 12/12 (1.0) | 0/12 (0.0) |
| Sow 5 (3) | 10/10 (1.0) | 10/10 (1.0) | 0/10 (0.0) |
| Sow 6 (3) | 9/12 (0.75) | 0/12 (0.0) | 0/12 (0.0) |
| Sow 7 (1) | 11/11 (1.0) | 11/11 (1.0) | 7/11 (0.64) |
| Sow 8 (1) | 14/14 (1.0) | 12/14 (0.86) | 0/14 (0.0) |

**Supplementary Table S2.** PRRSv vaccine responses in piglets born from sixteen PRRSv-vaccinated sows originating from two Belgian farrow-to-finish herds. For each sow the ELISA 1 (IDEXX ELISA), ELISA 2 (CIVTEST ELISA), VN (virus neutrlization assay) and PCR response ratio was calculated by dividing the number of PRRSv vaccinated, responding piglets by the total number of PRRSv-vaccinated piglets originating from each respective sow, at 3 weeks post-vaccination. Piglets of herd 1 were intramuscular PRRSv-vaccinated with the Porcilis® vaccine at 3 weeks of age. Piglets of herd 2 were intramuscular PRRSv-vaccinated with the Unistrain® vaccine at 4 weeks of age.

| Sow number (parity) | Response ratio ELISA 1 | Response ratio ELISA 2 | Response ratio VN | Response ratio PCR |
|---|---|---|---|---|
| *Herd 1* | | | | |
| Sow 1 (7) | 5/5 (1.0) | 5/5 (1.0) | 0/5 (0.0) | 5/5 (1.0) |
| Sow 2 (4) | 4/6 (0.67) | 4/6 (0.67) | 0/6 (0.0) | 4/6 (0.67) |
| Sow 3 (7) | 10/10 (1.0) | 6/10 (0.6) | 0/10 (0.0) | 10/10 (1.0) |
| Sow 4 (5) | 3/7 (0.43) | 3/7 (0.43) | 0/7 (0.0) | 3/7 (0.43) |
| Sow 5 (1) | 4/8 (0.5) | 2/8 (0.25) | 0/8 (0.0) | 5/8 (0.63) |
| Sow 6 (6) | 0/6 (0.0) | 0/6 (0.0) | 0/6 (0.0) | 0/6 (0.0) |
| Sow 7 (5) | 1/5 (0.2) | 0/5 (0.0) | 0/5 (0.0) | 1/5 (0.2) |
| Sow 8 (1) | 4/9 (0.44) | 0/9 (0.0) | 0/9 (0.0) | 5/9 (0.56) |
| | | | | |
| *Herd 2* | | | | |
| Sow 1 (5) | 6/6 (1.0) | 3/6 (0.5) | 0/6 (0.0) | 3/6 (0.5) |
| Sow 2 (3) | 3/5 (0.6) | 1/5 (0.2) | 0/5 (0.0) | 3/5 (0.6) |
| Sow 3 (3) | 7/9 (0.78) | 2/9 (0.22) | 0/9 (0.0) | 7/9 (0.78) |
| Sow 4 (4) | 8/8 (1.0) | 6/8 (0.75) | 1/8 (0.13) | 5/8 (0.63) |
| Sow 5 (3) | 4/6 (0.67) | 0/6 (0.0) | 0/6 (0.0) | 2/6 (0.33) |
| Sow 6 (3) | 6/8 (0.75) | 2/8 (0.25) | 0/8 (0.0) | 2/8 (0.25) |
| Sow 7 (1) | 0/7 (0.0) | 0/7 (0.0) | 0/7 (0.0) | 1/7 (0.14) |
| Sow 8 (1) | 6/10 (0.6) | 1/10 (0.1) | 0/10 (0.0) | 2/10 (0.2) |

**Supplementary Table S3.** PRRSv vaccine responses in piglets born from sixteen PRRSv-vaccinated sows originating from two Belgian farrow-to-finish herds. For each sow the ELISA 1 (IDEXX ELISA), ELISA 2 (CIVTEST ELISA), VN (virus neutrlization assay) and PCR response ratio was calculated by dividing the number of PRRSv vaccinated, responding piglets by the total number of PRRSv-vaccinated piglets originating from each respective sow, at 8 weeks post-vaccination. Piglets of herd 1 were intramuscular PRRSv-vaccinated with the Porcilis® vaccine at 3 weeks of age. Piglets of herd 2 were intramuscular PRRSv-vaccinated with the Unistrain® vaccine at 4 weeks of age.

| Sow number (parity) | Response ratio ELISA 1 | Response ratio ELISA 2 | Response ratio VN | Response ratio PCR |
|---|---|---|---|---|
| *Herd 1* | | | | |
| Sow 1 (7) | 5/5 (1.0) | 4/5 (0.8) | 3/5 (0.6) | 4/5 (0.8) |
| Sow 2 (4) | 5/6 (0.83) | 5/6 (0.83) | 5/6 (0.83) | 2/6 (0.33) |
| Sow 3 (7) | 9/10 (0.9) | 9/10 (0.9) | 3/10 (0.3) | 6/10 (0.6) |
| Sow 4 (5) | 4/7 (0.57) | 4/7 (0.57) | 3/7 (0.43) | 2/7 (0.29) |
| Sow 5 (1) | 5/8 (0.63) | 5/8 (0.63) | 2/8 (0.25) | 3/8 (0.38) |
| Sow 6 (6) | 0/6 (0.0) | 0/6 (0.0) | 1/6 (0.17) | 0/6 (0.0) |
| Sow 7 (5) | 4/5 (0.8) | 4/5 (0.8) | 0/5 (0.0) | 3/5 (0.6) |
| Sow 8 (1) | 7/9 (0.78) | 5/9 (0.56) | 2/9 (0.22) | 2/9 (0.22) |
| | | | | |
| *Herd 2* | | | | |
| Sow 1 (5) | 6/6 (1.0) | 6/6 (1.0) | 4/6 (0.67) | 5/6 (0.83) |
| Sow 2 (3) | 5/5 (1.0) | 4/5 (0.8) | 1/5 (0.2) | 4/5 (0.8) |
| Sow 3 (3) | 9/9 (1.0) | 9/9 (1.0) | 3/9 (0.33) | 8/9 (0.89) |
| Sow 4 (4) | 8/8 (1.0) | 8/8 (1.0) | 2/8 (0.25) | 7/8 (0.88) |
| Sow 5 (3) | 6/6 (1.0) | 5/6 (0.83) | 1/6 (0.17) | 4/6 (0.67) |
| Sow 6 (3) | 8/8 (1.0) | 8/8 (1.0) | 4/8 (0.5) | 8/8 (1.0) |
| Sow 7 (1) | 5/7 (0.71) | 6/7 (0.86) | 1/7 (0.14) | 7/7 (1.0) |
| Sow 8 (1) | 8/10 (0.8) | 8/10 (0.8) | 1/10 (0.1) | 10/10 (1.0) |
